# The Swedish monitoring of surface waters: 50 years of adaptive monitoring

**DOI:** 10.1007/s13280-014-0558-z

**Published:** 2014-11-15

**Authors:** Jens Fölster, Richard K. Johnson, Martyn N. Futter, Anders Wilander

**Affiliations:** Department of Aquatic Sciences and Assessment, Swedish University of Agricultural Sciences, P.O. Box 7050, 750 07 Uppsala, Sweden

**Keywords:** Acidification, Aquatic biology, Eutrophication, Freshwater, Time series, Water chemistry

## Abstract

For more than 50 years, scientific insights from surface water monitoring have supported Swedish evidence-based environmental management. Efforts to understand and control eutrophication in the 1960s led to construction of wastewater treatment plants with phosphorus retention, while acid rain research in the 1970s contributed to international legislation curbing emissions. By the 1990s, long-time series were being used to infer climate effects on surface water chemistry and biology. Monitoring data play a key role in implementing the EU Water Framework Directive and other legislation and have been used to show beneficial effects of agricultural management on Baltic Sea eutrophication. The Swedish experience demonstrates that well-designed and financially supported surface water monitoring can be used to understand and manage a range of stressors and societal concerns. Using scientifically sound adaptive monitoring principles to balance continuity and change has ensured long-time series and the capability to address new questions over time.

## Introduction

Human activity has adversely affected the quality and ecological integrity of surface waters on a global scale. Excessive use of phosphorus led to eutrophication and anoxia in many lakes around the world (Smith and Schindler 2008). The widespread use of nitrogen fertilizers in the 20th century resulted in eutrophication and subsequent anoxia in coastal receiving waters (Galloway et al. 2008). Since the 1970s, long-range transport of acidifying and toxic compounds has become a major cause of societal concern.

In 19th century, in Europe, urbanization made many surface waters unfit for human consumption. Early industrialization and inadequate infrastructure often caused local deterioration of water quality, leading to public criticisms (Chapra 2011). During the accelerated industrialization in Europe after the Second World War, many environmental problems escalated from merely local to regional and national scales. In the 1950s, several local and regional monitoring programs were initiated in Sweden to help identify and mitigate the impacts of waste water from factories and municipal sewage and other pressures on receiving waters (SEPA 1990). These early monitoring programs were restricted to only a few variables related to the specific form of pollution. At the same time, the Swedish Natural Science Research Council emphasized the need for underpinning science when addressing the deterioration of natural resources resulting from increased human impact (Willén 2001). Systematic investigations of aquatic ecosystems had been made by the scientific community since the beginning of the 20th century in Sweden. Taking a naturalist’s approach, the scope of this pioneering work was primarily to quantify and systematize nature. For example, Eriksson (1929) measured major solutes in a large number of streams between 1909 and 1925, with the aim of quantifying the transport of weathering products from land to sea by surface waters, while between 1929 and 1937, Lohammar (1938) studied relationships between macrophytes and water chemistry in lakes in central and northern Sweden. Today, these and other early works act as valuable historical references.

**Fig. 1:**
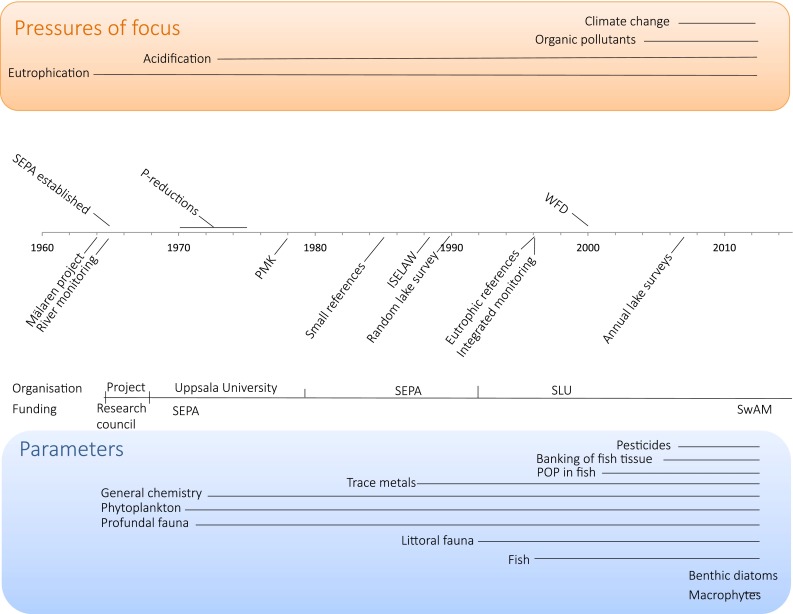
Development of the Swedish national surface water monitoring program in response to environmental pressures. During the 1960s, monitoring developed from a research project to a government-financed program runs at Uppsala University. During the 1970s, the focus was broadened from eutrophication toward acidification. Further, surface waters were incorporated into a national monitoring program for all ecosystems (PMK), and the monitoring group and laboratories were organizationally moved to the Swedish Environmental Protection Agency (SEPA), although co-located with the Limnology department at Uppsala University. During the 1980s, the focus on acidification was reinforced by monitoring of small reference lakes and streams, as well as expanding the parameter list with trace metals and littoral fauna. In 1989, a monitoring program focused on limed lakes and streams started (ISELAW). Further lake surveys based on a random selection were performed since 1990. Fish monitoring, performed by the Fisheries Board, was integrated into the ISELAW program in 1994 and other national surface water monitoring programs in 1997. Problems with persistent organic pollutants (POP) had been on the agenda since the early 1960’s, but it was not until 1997 that POPs were included in the surface water monitoring program. The acidification focus was further strengthened by the start of the new program of integrated monitoring of small catchments. In the year 2000, the EU Water Framework Directive (WFD) was ratified. This led to the development of biological monitoring and the inclusion of more sites and the addition of benthic epiphytes and macrophytes to the parameter list. As acid deposition declined markedly, focus of the monitoring in the small lakes and streams as well as the integrated monitoring shifted toward climate change, where the long-time series have shown to be extremely valuable. In 2011, the responsibility of the surface water monitoring was changed from SEPA to a new authority SwAM (The Swedish Agency for Marine and Water Management)

In 1964, efforts by the Swedish Natural Science Research Council led to the investigation of Mälaren, the third largest lake in Sweden with a large population living within its catchment. The scope of the investigation was to better understand the on-going eutrophication of the lake. Parallel to this study, a river monitoring program was initiated between 1962 and 1965, and the Swedish Natural Science Research Council funded an expansion of the program including *inter alia* greater spatial coverage and a wider range of chemical parameters, including nutrients. When the Swedish Environmental Protection Agency (SEPA) was established in 1967, these two programs became the core of a gradually expanding national monitoring program of fresh water quality in Sweden (Fig. 1). Today this program comprises regular long-term monitoring of water chemistry and biodiversity in 114 streams and 110 lakes, and a probability-sampling program includes 4800 lakes. The strong link between monitoring, the broader scientific community, and the regulatory authorities has continued since the start of the programs and has been vital to the accrual of high-quality data of societal relevance.

A number of studies have noted importance of long-time series for successful environmental management (e.g. Lindenmayer and Likens 2009, 2010; Burt et al. 2011; Howden et al. 2011). Often 12 years or more years of data are needed to separate human-induced trends from natural between-year variability (Howden et al. 2011), and time series of several decades can be needed to address long-term climatic control on water quality in relation to anthropogenic trends (Erlandsson et al. 2008). Further, long-time series are often critical for detecting abrupt changes of water quality resulting from catchment land use (e.g., changes resulting from increased human pressure), especially in high-latitude countries such as Sweden (Tetzlaff et al. 2013).

The value of long-time series has to be balanced to the need for adapting monitoring programs to meet new societally driven questions and to adopt new knowledge and techniques, when pertinent. Such developments should be based on the following criteria for adaptive monitoring: (i) well-formulated, tractable questions (ii) on-going development of new questions as old questions are answered or important new questions arise; (iii) robust experimental design; (iv) well-formulated protocols for data collection and sample handling; (v) collaborative partnerships among scientists, responsible agencies, and other stakeholders; (vi) an information system which makes the data available to interested parties; (vii) widespread use of the data; (viii) secure long-term funding, and (ix) strong and enduring leadership (Lindenmayer and Likens 2009, 2010).

Here, we review the development of the Swedish long-term surface water monitoring program. First, we describe how adaptive monitoring was implemented to focus on societally relevant questions related to eutrophication and acidification, as well as national and European environmental policies. Then we describe today’s national fresh water monitoring program. Finally, we discuss future challenges for the monitoring program and how they could be met.

## The investigation of water quality in lake Mälaren

The most apparent effect on the environment from improved standards of living in mid-20th century Sweden and elsewhere was lake eutrophication caused by point-source pollution associated with insufficient wastewater treatment. Massive algal blooms in Mälaren and subsequent hypoxia affected drinking water supply, fisheries and caused deep concern to the population living close to the lake. Mälaren is a regionally important lake that provides drinking water and recreation to 1.5 million people (Köhler et al. 2013). The lake became the focus of the first comprehensive limnological investigation in Sweden including both physiochemical and biological parameters (Willén 2001). The investigation was not only restricted to the lake itself, but also included tributaries to allow for regional-scale quantification of the total input of nutrients to the lake, including those from diffuse agricultural and other non-point sources. Studies in Mälaren were followed by investigations of the other large lakes in Sweden: Hjälmaren (1965), Vättern (1966), and Vänern (1971). When the newly established SEPA assumed responsibility for these lakes in 1967, the studies became part of a continuous monitoring program, comprising water chemistry, phytoplankton, zooplankton, and profundal macroinvertebrates. The main focus of these studies was to elucidate the importance of phosphorus from wastewater as a driver of poor water quality. Having established scientifically sound links between excess wastewater phosphorus and algal blooms, it was possible (in 1969) to introduce efficient wastewater treatment, including phosphorus stripping in all larger wastewater treatment plants.

**Fig. 2:**
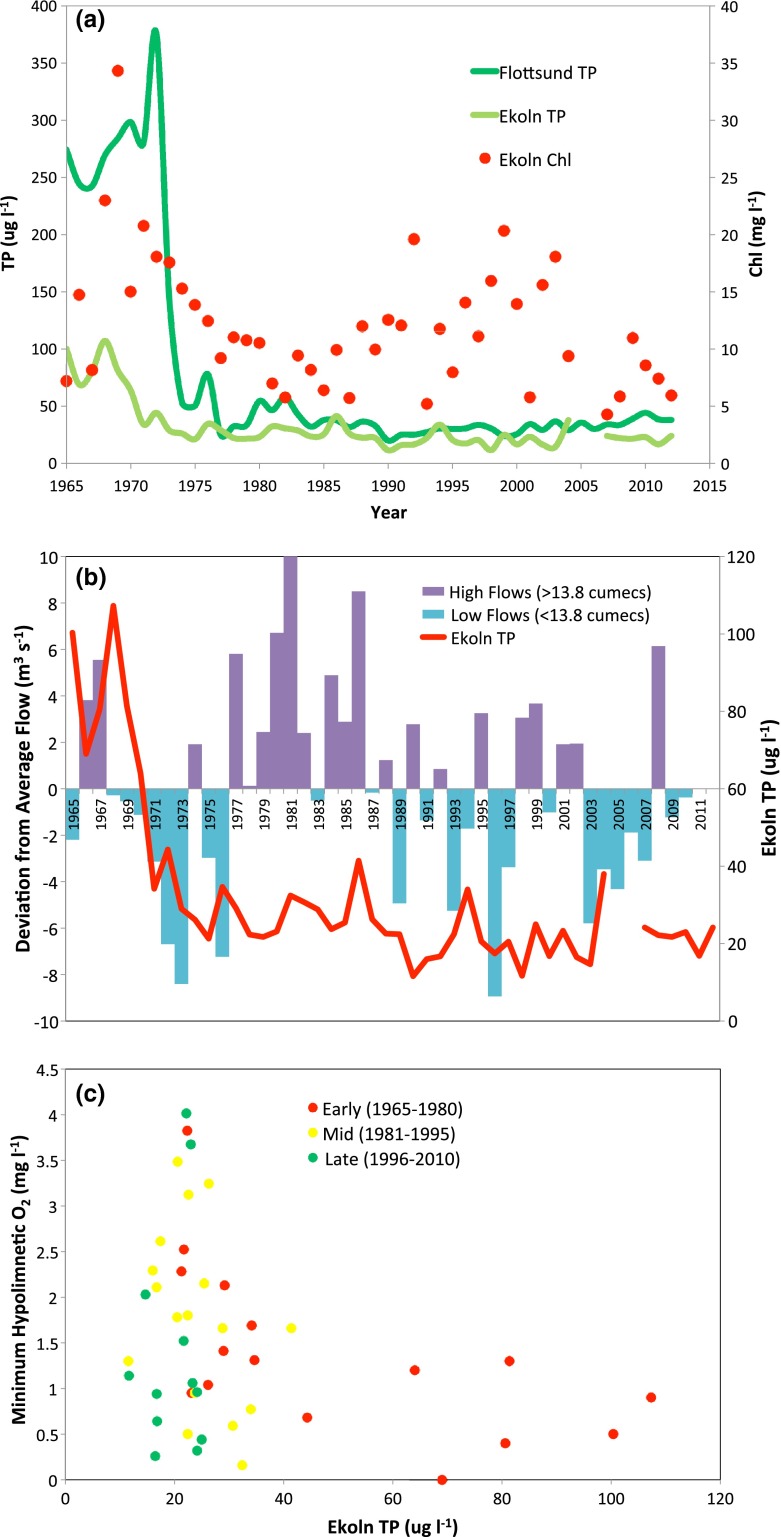
Recovery from eutrophication in Ekoln after introduction of improved wastewater phosphorus removal in 1973. **a** Total phosphorus (TP) concentrations at Flottsund in the Fyrisån immediately downstream of the wastewater treatment plant and TP and chlorophyll at Ekoln, in Mälaren near the Fyrisån outlet. **b** Annual average Fyrisån flows and Ekoln TP. **c** Ekoln summer hypolimnetic oxygen concentrations versus average annual TP. Ekoln bottom waters still experience periods of oxygen stress (**c**), and an inverse relationship exists between TP and hypolimnetic oxygen minima. As long as average TP exceeded about 40 µg L^−1^, minimum hypolimnetic oxygen concentrations remained below 2 mg L^−1^. In recent years, there has been no clear relationship between average TP and minimum oxygen concentrations. Lower TP concentrations at Ekoln sometimes but not always result in hypolimnetic oxygen concentrations more likely to support viable biological communities

The beneficial effects of this measure were apparent almost immediately. Total phosphorus (TP) concentrations at Flottsund in the Fyris River immediately downstream of the Uppsala wastewater treatment plant responded rapidly to the introduction of phosphorus stripping in 1973 (Fig. 2a). Declines in TP concentration at Ekoln, a bay in Mälaren near the outlet of the Fyris, were less pronounced. Chlorophyll concentrations in Ekoln declined during the late 1970s and early 1980s, as would be expected with a decline in TP (Fig. 2a). Between the mid 1980s and today, chlorophyll concentrations have been variable, suggesting the importance of other factors controlling algal development. Interpretation of TP trends associated with wastewater phosphorus removal was complicated by interannual variability in flows (Fig. 2b) and internal processing in the lake (Fig. 2c).

The early 1970s was an unusually dry period in central Sweden, and river flows were well below average, leading to lower than normal diffuse phosphorus inputs. Combined, these reductions in phosphorus loads resulted in improvements in water quality (e.g. increased Secchi depth transparency) far above expectations. Secchi depth transparency, far above expectations (Wilander and Persson 2001; Willén 2001). Nonetheless, rapid improvement in water quality after the introduction of improved wastewater treatment helped to encourage a positive attitude in Swedish society for further environmental management efforts.

The time series from Ekoln show the value of long-term, multi-site monitoring. The TP monitoring at Flottsund, immediately downstream of the Uppsala wastewater treatment plant, was necessary for regulatory purposes to confirm the efficacy of measures to limit phosphorus loading to surface waters. The monitoring at Ekoln in the lake Mälaren showed that TP did not respond as rapidly as found at Flottsund. Regional monitoring is important as it places regulatory monitoring into context. Longer-term monitoring increases the likelihood that causal relationships can be correctly identified. Short-term monitoring might have over-estimated the efficacy of improve phosphorus removal during wastewater treatment and would have failed to identify the magnitude of natural variability. Multi-parameter monitoring is needed to link the efficacy of management intervention to ecological response.

Monitoring of Mälaren is on-going (Sonesten et al. 2013) and provides an important resource for understanding the combined effects of changes in climate, land use, and post-glacial rebound on water quality.

## Development of regional lake surveys and river monitoring

Key elements of the evolving Swedish surface water monitoring program included on-going investigations of Sweden’s four largest lakes and initiation of water chemistry monitoring in rivers mouths and upstream sites (Wiederholm and Johnson 1997). The main monitoring goals were to adequately characterize unperturbed reference conditions (Odén and Ahl 1970; SEPA 1980), to estimate riverine fluxes to the sea and to better understand the role of catchment processes controlling element fluxes. The aim of the river monitoring program was to quantify the transport of both pollutants and natural substances to the large lakes and surrounding seas. The ambition to quantify the transport of natural substances dates back to pioneering work by Eriksson (1929), and some of the sites in the new monitoring program are adjacent to his study sites. The inclusion of reference sites and a wide range of physiochemical parameters were seen as important steps for separating the effects of human-induced changes from natural variation. The program was gradually expanded to include more sites and currently comprises 47 river mouths and ten upstream reference sites. The river mouth monitoring plays a key role in estimating nutrient loads to the marine environment and for reporting to the Baltic Marine Environment Protection Commission-Helsinki Commission (HELCOM) (Brandt et al. 2009).

To provide a spatially extensive assessment of the physicochemical status of lakes in general and the widespread effects of eutrophication in particular, the first national lake survey including 1250 lakes distributed across the entire country was performed in August 1972 (Johansson and Karlgren 1974). The lakes were selected by local county boards, providing a geographically representative coverage of the lake types within each county. Some counties used statistical approaches to select the lakes, but most selections were based on subjective criteria and therefore cannot be regarded as representative for the whole lake population (Dietrichsson 1975a).

## Acidification leads to further expansion of the monitoring program

In 1967, regional acidification of freshwaters was revealed as new environmental threat by Svante Odén who first hypothesized that fish death in lakes situated on the Swedish west coast were related to decreasing pH in precipitation over northern Europe (Odén and Ahl 1976). Recognition of anthropogenic acidification as a driver of changing water quality required a change in focus from local (often point sources) pollution to an international scale and consequent adaptations to the monitoring program. Most of the pollutants deposited over Sweden were assumed to originate from elsewhere in Europe. The concept of long-range transboundary pollution was raised by Scandinavia at an international level in order to persuade other countries to reduce their acidifying emissions. Data from the Scandinavian surface water monitoring programs were used to underpin findings, but interpretation of the temporal variation in time series data was not always straightforward. For example, at the UN conference on the human environment in 1972, time series data from large rivers showed decreasing trends in pH between 1965 and 1971. An abrupt increase in pH in winter 1969–1970 was interpreted as a temporary increase due to unusual weather conditions that was expected to be followed by a return to acidification (Odén and Ahl 1976). Later, the decrease in pH between 1965 and 1971 was shown to be correlated with climatic variation, i.e., due to natural variation. As with the unexpected recovery from eutrophication in Mälaren, where both reduced loading from wastewater treatment plants and drier years resulted in lower levels of diffuse inputs, this example was another lesson that temporal patterns should be interpreted with caution and that long-time series of a decade or longer are needed to determine causality. Long-term monitoring of unperturbed reference sites has also been shown to be invaluable for distinguishing natural variation from anthropogenic change.

Over time, results from the monitoring program showed that acidification affected mostly smaller catchments and not the larger, better buffered, rivers. To better understand the widespread effects of acidification, the lake survey was repeated in 1975; however, this time the survey was performed in spring when pH was expected to be at a minimum (Dietrichsson 1975b). Lake surveys with a focus on acidification were repeated every fifth year from 1985 until 2005, thereby providing an important contribution to regional acidification assessments (Henriksen et al. 1998; Skjelkvåle et al. 2001). In 1985 and 1990, samples were taken from ice during winter for practical reasons, but in 1995, the use of helicopters enabled sampling during autumn circulation, thereby providing a more representative sample of the whole lake (Göransson 2003). In 1995 and 2000, littoral macroinvertebrates were sampled from a subset of lakes (*n* = 700) and streams (*n* = 700) to determine the effects of acidification and other pressures on biological integrity (Wilander et al. 1998, 2003). Since 2007, all lake surveys have been conducted annually with one-sixth of the total of 4800 lakes sampled each year (see below). The new layout accounted for the interannual variation in water quality and was more stably funded.

In 1982, an extensive national monitoring program was started to better understand the long-term effects of airborne pollutants (acidification) on lakes and streams. A spatially extensive network comprising 190 reference lakes was monitored by the end of the decade, and most of these sites remain as part of national or regional monitoring programs. Monitoring consisted of surface water chemistry (four times per year), and in a subset (*n* = 26) of the lakes, biological monitoring of fish, benthic macroinvertebrates, phytoplankton, and zooplankton was conducted annually (SEPA 1985). Furthermore, 12 small forest streams were monitored for water chemistry and benthic macroinvertebrates (riffle assemblages). Situated in small catchments within nature reserves, these streams were also part of an integrated monitoring program including measures of meteorological, physiochemical, and biological parameters in aquatic and terrestrial habitats (SEPA 1985). Since 1978, national monitoring of surface waters has been included in a comprehensive national monitoring program (at the time called “Program för övervakning av miljökvalitet”, PMK) including air quality, terrestrial ecosystems, ground water, fresh water, coastal waters, and marine water financed by SEPA (SEPA 1980). To better understand the long-term, mitigating effects of liming on physicochemical and biological integrity of acidified lakes and streams, monitoring of 14 limed lakes and 7 limed streams was started in 1989 (Appelberg and Svenson 2001). A wide range of physicochemical and biological variables (fish, macroinvertebrates, phytoplankton, and zooplankton) was included in this monitoring program. However, one of the main shortcomings of this program is the lack of measurements taken prior to liming.

In summary, by the end of 1980s, the national inland surface water monitoring program comprised monthly water chemistry sampling in 49 river mouths and 39 upstream sites as well as annual monitoring of surface water quality and biology in four of Sweden’s largest lakes and their tributaries. Furthermore, the program included the newly implemented, spatially extensive (by survey and annual monitoring), and temporally intensive monitoring of time series reference lakes and streams and limed lakes and streams. The Swedish national monitoring of freshwater had a very high ambition level, largely reflecting the prominence of environmental issues on the political agenda at the time. It was also related to the fact that monitoring data had played a key role in reaching agreements about reductions in acid deposition and assessing the efficacy of programs to curb eutrophication. An evaluation of the Swedish monitoring program by an international expert panel in 1986 concluded that the program was unique in many respects. Unlike many monitoring programs (reviewed in Lovett et al. 2007; Lindenmayer and Likens 2009, 2010), the Swedish program was cost-efficient, focused on important environmental issues, and well integrated with the broader scientific community (SEPA 1986). Moreover, the evaluators stated that the program had good cooperation with other international monitoring programs, although it was deemed to be more developed and versatile than many other programs. Indeed, the newly established integrated monitoring of reference sites was especially praised. Suggestions for improvement focused on inclusion of biological variables, such as benthic macroinvertebrates and fish in the time series and survey monitoring programs of lakes and streams, as well as measurements of heavy metals and organic pollutants. Further, it was recommended to reduce, if possible, the number of sites following a statistical evaluation of the data.

## The 1996 revision

**Fig. 3:**
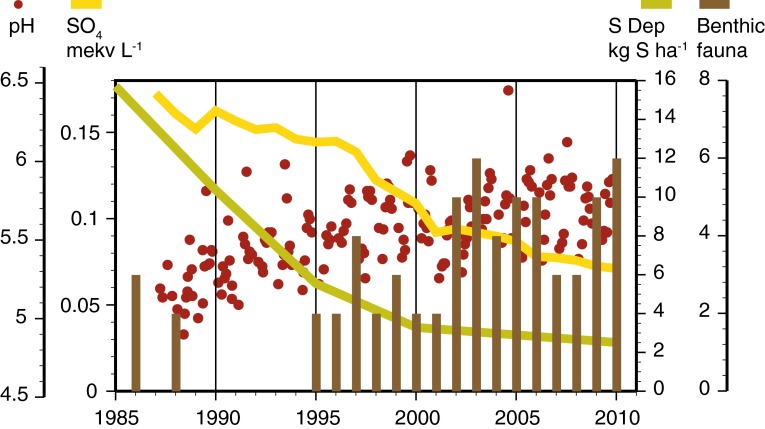
Recovery of the acidified lake Övre Skärsjön showing trends in sulfate deposition, in-lake sulfate concentration, pH, and the Henriksen–Medin benthic community acidification index. Since the mid 1980s, declines in S deposition have been reflected in declining in-lake sulfate concentration and increasing pH. The decline in-lake sulfate concentration is less than that in deposition due to soil retention processes. Between 1987 and 2010, pH has increased by ~1 unit, which is slightly larger than within year variation. This pH increase is associated with biological community improvements where Henriksen–Medin index values have increased since the mid 1980s. These results show the value of complementary biological and chemical data for assessing the efficacy of emissions control scenarios. Increasing pH and improvements in biology can be unequivocally linked to declining acid deposition, but significant within year variation in pH which shows the need for frequent monitoring to correctly identify both trends and variability

In revising the national monitoring program in 1996, some recommendations including biological monitoring (benthic macroinvertebrates and fish) and analysis of selected trace metals were immediately implemented. The suggestions were further developed by a working group that led to a revision of the program in 1996 (SEPA 1992). The structure of the revised program included three tiers of monitoring with (i) statistically representative periodic surveys to monitor large-scale changes in surface water quality, (ii) annual monitoring of some 100 lakes and 50 streams to address changes in surface water quality and biological integrity, and (iii) intensive monitoring of a subset of time series lakes and streams to address terrestrial (catchment)—aquatic linkages. An example of the benefit of integrated ecosystem monitoring of lakes is shown in Fig. 3, where the recovery from acidification can be followed both by chemical as biological parameters. The lake surveys in 1995 and 2000 included around 3000 lakes sampled during autumn circulation and littoral macroinvertebrates were sampled in a subset of 700 lakes. Lakes were selected by a stratified random selection from a national lake register comprising 85 000 lakes. Although the lake register included lakes as small as 1 ha, the survey was restricted to lakes >4 ha to harmonize with other Nordic monitoring programs (Henriksen et al. 1998). The chemical and biological lake surveys and an additional survey of benthic macroinvertebrates and water chemistry from 700 intermediate-sized streams were performed to quantify the acidification status of lakes and to provide data for estimating critical loads for acidification within the UN-ECE Air Convention on Long-Range Transboundary Air pollution.

The number of lakes monitored annually was decreased from 190 to about 100. Most of the retained lakes were a subset of the previous time series program, but some humic lakes were included to improve the representativeness of the monitoring program based on a statistical evaluation of the data (Johnson 1999). Furthermore, a few naturally eutrophic lakes were added as the overall objective of the monitoring program was extended from assessing water quality effects of airborne pollutants to assessing impacts of changes in land use (such as agriculture). Monitoring of many of the lakes excluded from the national monitoring program has continued with support from regional monitoring boards. The revised program also included the monitoring of four headwater catchments as part of an integrated, biogeochemical catchment-modelling program (Starr 2011). This research field that has grown in the last decade and shown to be a powerful tool in linking airborne pollution and a changing climate with soil and water quality (Moldan and Cerny 1992). Also as part of the 1996 revision, measurements were made of POPs (persistent organic pollutants) in samples of fish tissue, which were later archived to allow for determination of new substances in the future (Nyberg et al. 2014).

As part of the 1996 revision, responsibility for monitoring the four largest lakes and their tributaries was transferred from national (SEPA) to regional organizations (i.e., drainage area based). One result of this organizational move is that a suite of consultant agencies have been responsible for implementing the monitoring programs; with varying results, changes in data quality, and discontinuities in the time series, which has made it more difficult to separate the effects of changing laboratories from trends in the environment (Holmborn et al. 2011).

## The EU water framework directive, wfd

In 2000, the EU parliament adopted the Water Frame Directive (WFD), which has strongly influenced monitoring and management of freshwater and coastal habitats across Europe (EC 2000). The primary objective of the directive is that all water bodies should achieve “good ecological status”, defined as a minor deviation only from a reference state with negligible disturbance from human impact, by 2015. Implementation of the WFD by member states has resulted in a shift in water management to catchment-based monitoring and restoration, with greater focus on aquatic ecology and less on surface water quality (Hering et al. 2010).

Monitoring within the WFD includes surveillance, operational, and investigatory monitoring (EC 2000). Most national programs conduct surveillance monitoring to support WFD status classification and to provide information for designing efficient investigatory monitoring programs, to detect long-term changes in natural systems and long-term changes in large-scale human impact (EC 2000, Annex 5). The large numbers of long-term lake and stream time series in the Swedish national monitoring program are extremely valuable for fulfilling the two latter aims of WFD monitoring. The reference lakes and streams have also been a part of the network of sites representative of different water body types needed to define reference conditions, as required by the WFD. Swedish reference sites have also been used for inter-calibration of northern continental Europe, where suitable reference sites are often difficult to find (Järvinen et al. 2013). The lake water chemistry survey based on stratified random selection has the potential to form the basis for a cost-efficient monitoring program by combining an impact analysis to identify water bodies at risk of not reaching good ecological status, so as to better target monitoring of lake biota.

The emphasis on biological monitoring by the WFD led to the introduction of epiphytic diatoms in streams as a new parameter in the Swedish national monitoring (Kahlert et al. 2006), and the establishment of new stream sites that were not affected by hydromorphological disturbance and harboring populations of migrating fish. In lakes, macrophytes were also introduced as a new parameter (Ecke 2007). The emphasis on hazardous substances has also highlighted the fact that fish from most lakes in Sweden have levels of mercury in excess of EU limits (Åkerblom et al. 2014).

## Today’s monitoring program

The Swedish national surface water monitoring program comprises a large number of lakes and streams to meet the information demands from the WFD as well as UN-ECE LRTAP, OSPAR, and HELCOM conventions and the national environmental goals. International standards when available are used in all sampling and analyses (Table 1).

**Table 1:** Standard methods for sampling of water chemistry and biological parameters in the Swedish national monitoring of surface water. For further details see http://www.slu.se/aquatic-sciences/waterlab

Sampling methods	Method
Water chemistry	SS-EN ISO 5667-1:2007
Trace metals	SS 02 81 94
Benthic fauna in profundal and sublittoral, grab sampler	SS 02 81 90
Benthic fauna, kick sample	SS-EN 27828
Phytoplankton	SS-EN 15204:2006
Zooplankton	SS-EN 15110:2006
Benthic diatoms	SS-EN 13946:2003
Fish, electrofishing	SS-EN 14011:2006
Fish gillnets	SS-EN 14757:2006

**Fig. 4:**
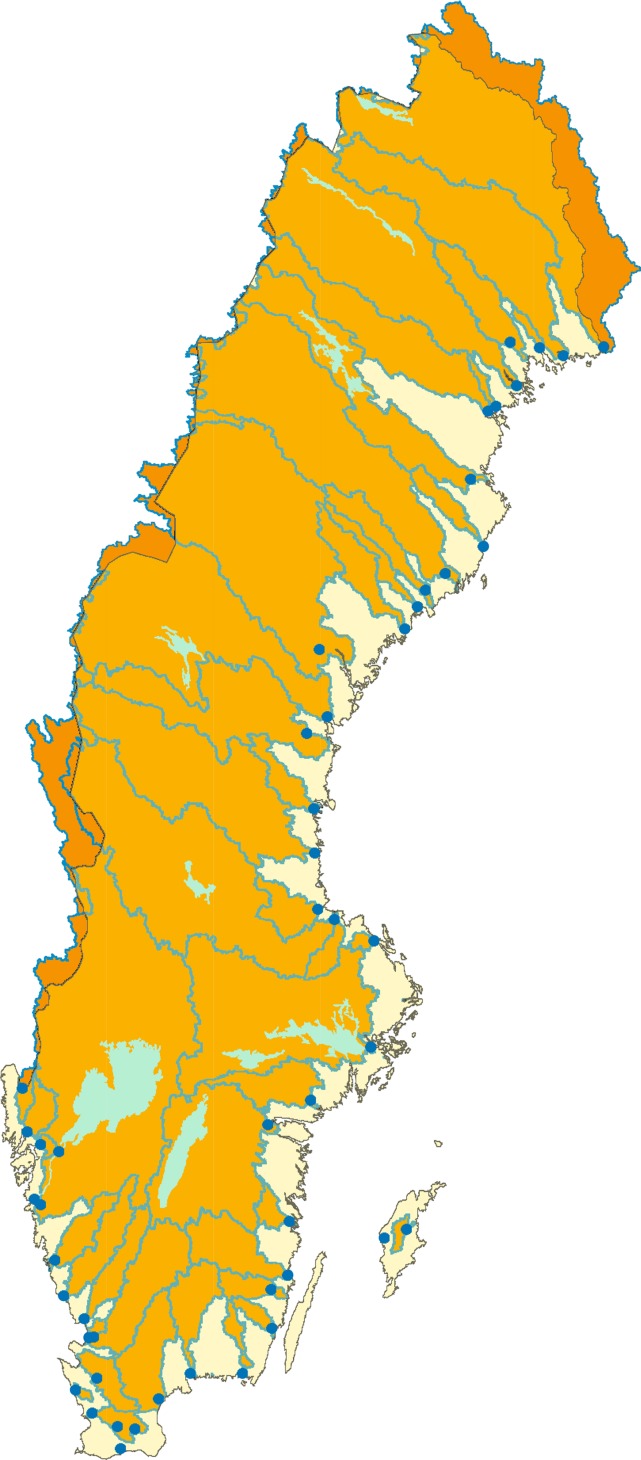
Monitored river mouths within the Swedish national monitoring program 2014 with catchment borders given. The 47 monitored rivers cover runoff from 82 % of the surface of Sweden. Bright color denotes non-monitored areas, and dark color denotes catchment areas outside Sweden (i.e., Norway and Finland)

The monitoring program has retained the three-tier nested design from 1996. The tier one lake survey program currently includes 4800 lakes sampled on a six-year cycle. Lakes were selected by a stratified random selection from the national lake register of all lakes >1 ha (Grandin 2007). Stratification was used to obtain a relatively larger number of lakes from southern Sweden, where environmental pressures are higher and to sample more larger lakes since a pure random selection would result in 96 % of the sampled lakes having a surface area <1 km^2^ (80 % of Swedish lakes are <10 ha). Water samples for analysis of chemistry are taken by helicopter during autumn circulation, between mid-September in the alpine region and mid-November in the southern coastal areas. Based on earlier experiences of trace metal contamination, helicopters with turbo jet (as opposed to piston) engines are used, and samples are taken using a tube sampler with no free metal surfaces. Statistical distributions of the chemical parameters analyzed for all lakes in Sweden were calculated by a re-stratification of the lake survey (Table 2). Tier 1 also includes 47 river mouths with continuous monthly sampling for water quality (Fig. 4). The monitored river mouths cover runoff from 82 % of the surface area of Sweden.

**Table 2:** Percentiles of chemical parameters in Swedish lakes >1 ha estimated by a destratification of the national lake survey 2007–2012. For trace metals from Cu and further down, data are from 2010 to 2012

Percentile	5	25	50	75	95
pH	5.1	6.2	6.7	7.0	7.5
Cond. mS m^−1^ (25 °C)	0.76	1.65	2.54	4.44	10.3
Ca meq L^−1^	0.022	0.07	0.129	0.232	0.679
Mg meq L^−1^	0.011	0.03	0.049	0.075	0.177
Na meq L^−1^	0.019	0.035	0.051	0.077	0.247
K meq L^−1^	0.003	0.006	0.009	0.015	0.034
Alkalinity^a^ meq L^−1^	−0.017	0.045	0.108	0.212	0.702
SO_4_ meq L^−1^	0.006	0.015	0.027	0.052	0.154
Cl meq L^−1^	0.008	0.013	0.021	0.05	0.247
F mg L^−1^	0.01	0.03	0.07	0.14	0.33
NH_4_-N µg L^−1^	1	4	8	22	105
NO_2_ + NO_3_-N µg L^−1^	1	1	5	19	104
Tot-N µg L^−1^	87	194	315	457	923
PO_4_-P µg L^−1^	1	1	2	3	7
Tot-P µg L^−1^	2	5	8	13	31
Absorbency_420 nm_^b^	0.006	0.044	0.116	0.252	0.571
Si mg L^−1^	0.1	0.7	1.7	2.9	4.5
TOC mg L^−1^	1.5	5.0	8.9	14.6	26.0
Al µg L^−1^	8.2	26	64	160	410
Fe µg L^−1^	10	51	230	740	2000
Mn µg L^−1^	0.43	2.5	11	32	100
Cu µg L^−1^	0.11	0.22	0.38	0.65	1.5
Zn µg L^−1^	0.3	0.77	1.4	2.8	6.3
Pb µg L^−1^	0.02	0.07	0.15	0.32	0.95
Cd µg L^−1^	0.0025	0.005	0.009	0.016	0.0488
Cr µg L^−1^	0.025	0.07	0.14	0.25	0.59
Co µg L^−1^	0.011	0.0297	0.062	0.146	0.517
Ni µg L^−1^	0.05	0.12	0.23	0.46	1.2
As µg L^−1^	0.03	0.08	0.19	0.39	1
V µg L^−1^	0.03	0.06	0.14	0.34	0.99

^a^Negative alkalinity denotes acidity

^b^Absorbency is measured on filtered samples in a 5 cm cell

**Fig. 5:**
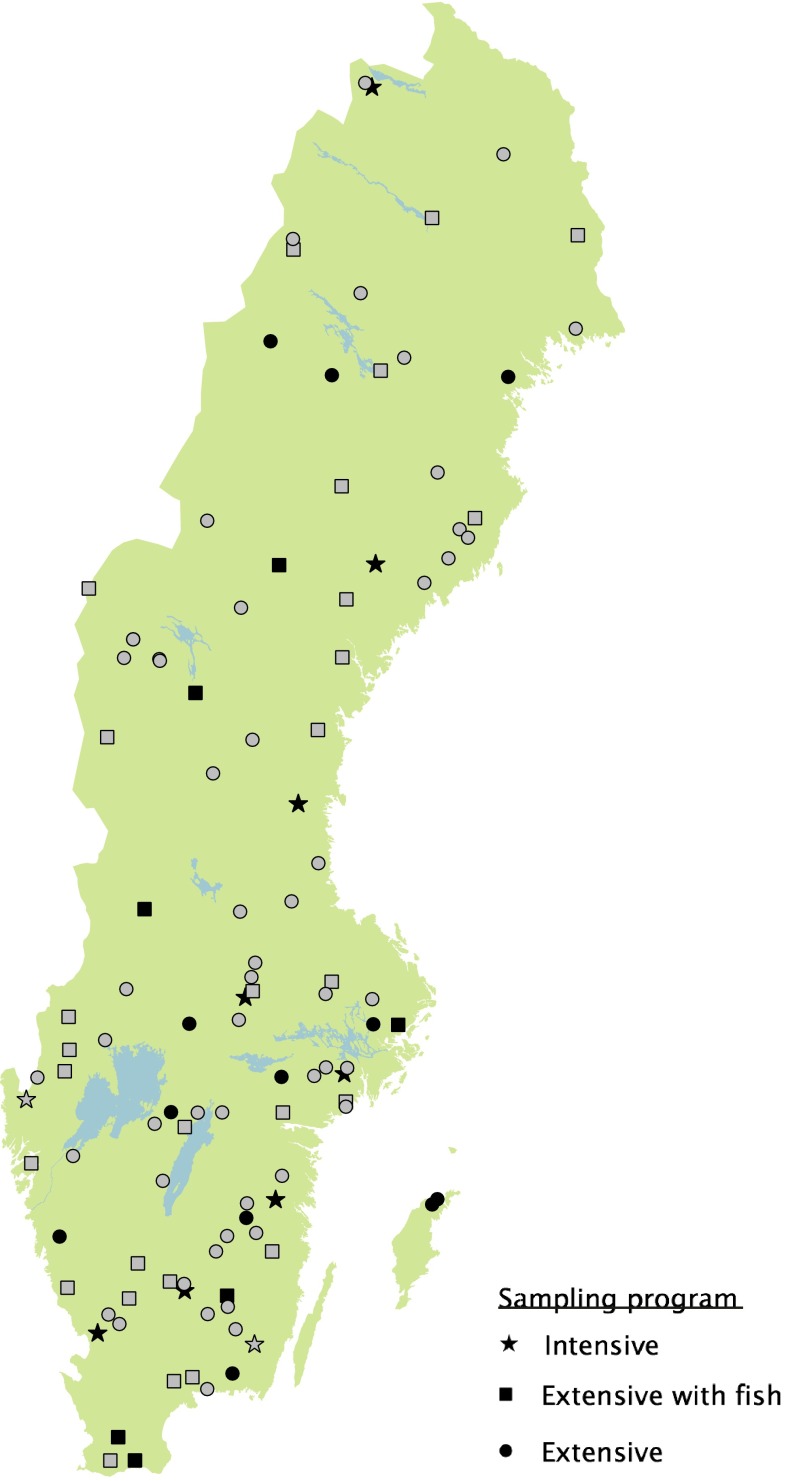
Trend lakes within the Swedish national monitoring program 2013. The form of the *symbol* denotes sampling program. *Black symbol* denotes banking of fish tissue. The trend lakes include 108 lakes with ten lakes more intensively monitored. The lakes are reference lakes in terms of point sources and intensive local land use, but may be affected by large-scale airborne pollution and extensive land use

**Fig. 6:**
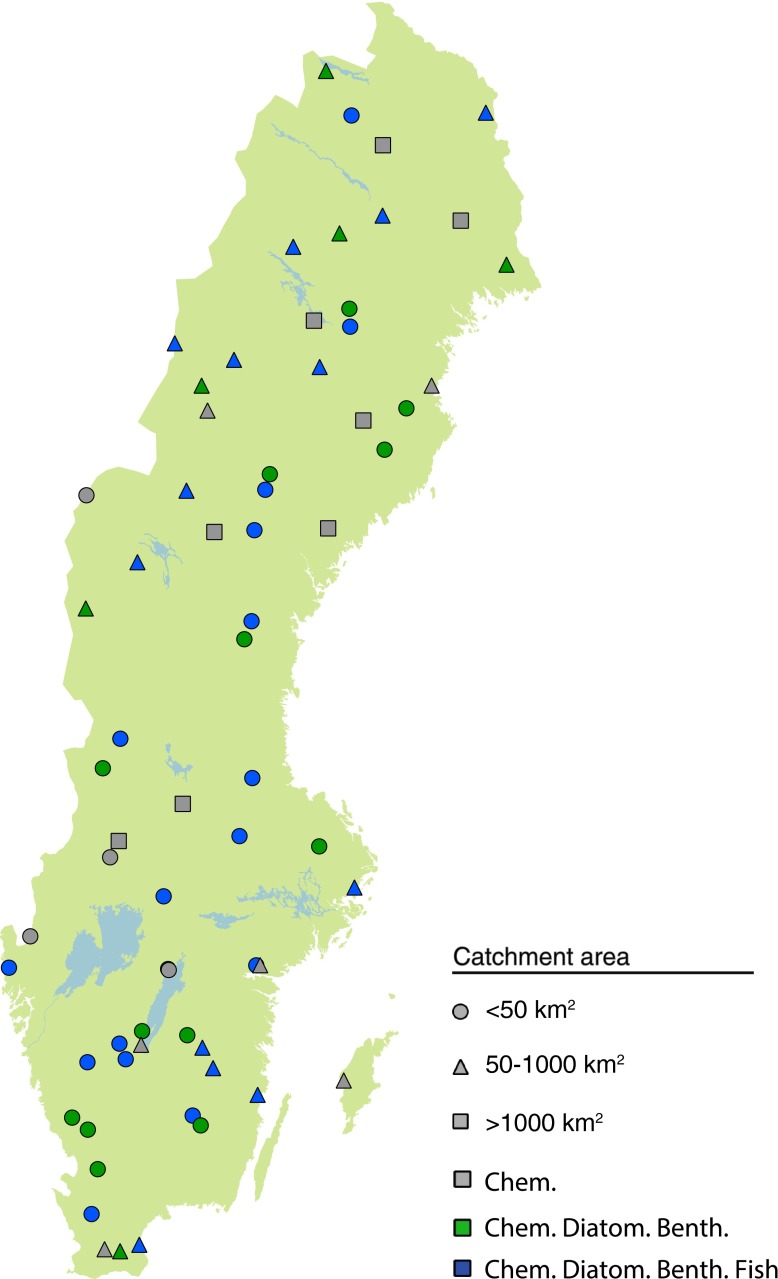
Monitored streams within the Swedish national monitoring program 2014. The form of the symbol denotes the catchment size, and color denotes the sampled parameters. The national program for trend streams includes 67 streams with catchment size between 1 and 10 000 km^2^. The 29 sites with all parameters are regarded as reference stations for defining reference conditions for ecological status. The other stations might be affected by diffuse pollution, acidification, or eutrophication

In the second tier of monitoring, time series sites are monitored every year within the programs “Trend Lakes” and “Trend Streams”. The ±100 time series lakes (Fig. 5) and 67 time series streams (Fig. 6) are small- to intermediate-sized water bodies with minimal influence from point sources; hence water quality is controlled primarily by climatic factors and long-range transboundary pollutants. The Trend Lake program consists of surface water chemistry four times per year, representing summer and winter stagnation and spring and autumn circulation periods. Littoral and profundal macroinvertebrates are sampled during autumn and phytoplankton during summer stagnation in August. Fish are sampled by standardized gill net every third year and macrophyte assemblages every sixth year. In 27 lakes, fish tissues are sampled and frozen (−30 °C) for future analysis. The Trend Stream program includes monthly water chemistry sampling in 67 streams and annual biological monitoring in a subset of 49 streams using benthic macroinvertebrates and epiphyton. In 29 streams, fish assemblages are monitored annually (by electrofishing).

**Fig. 7:**
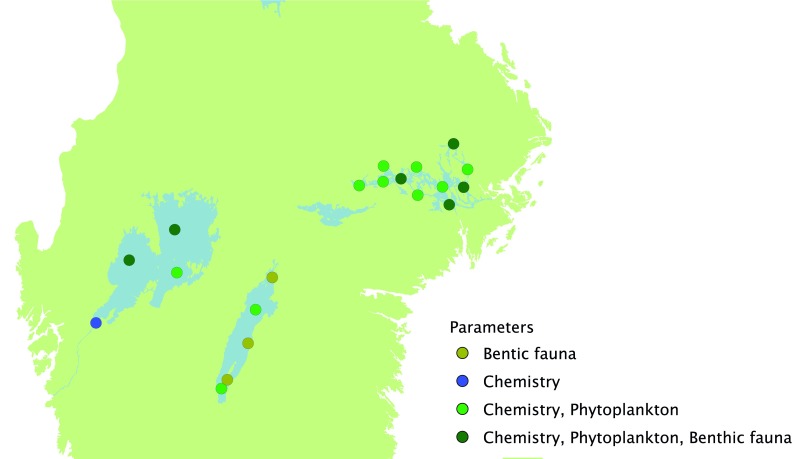
Monitored stations in the three large lakes Vänern, Vättern, and Mälaren within the national Swedish monitoring program

In the third tier, 10 of the second-tier monitoring time series lakes are more intensively sampled for water chemistry at three depths during the open water season (March to October/November). In the same lakes, fish and littoral, sublittoral, and profundal macroinvertebrate assemblages are sampled once per year; phytoplankton and zooplankton are sampled four times per year, while macrophytes are sampled approximately once every sixth year. The long-time series of chemical and biological data from these lakes are valuable for assessing recovery from acidification (Fig. 3). The program for integrated monitoring of four headwater catchments is also a part of tier three. One of the integrated monitoring sites was hit by a storm in 2005 which felled many trees in the catchment. Thus, the site was fortuitously turned into a platform for the ecosystem-scale study of the effects of extreme events (Löfgren et al. 2014). The national monitoring program of the three largest lakes Vänern, Vättern, and Mälaren currently consists of water quality and phytoplankton at 16 sites, zooplankton at 9 sites, profundal macroinvertebrates at 11 sites, and toxic substances in fish at 5 sites (Fig. 7).

## Data storage, use, and availability

Data have been stored electronically since the beginning of the monitoring program and have been freely available over the Internet since the late 1990s. Today data are available on http://miljodata.slu.se/mvm/.

The laboratories at the Department of Aquatic Sciences and Assessment have been certified by the Swedish Board for Accreditation and Conformity Assessment (SWEDAC) since 1992 for water chemistry and since 1995 for biology. All data are subject to extensive quality control check. Standard methods are used whenever possible, and any methodological changes are extensively scrutinized and documented. Over time, chemistry data have become more precise with less random fluctuation. Biological data have become more diverse over time as organisms have been identified to lower taxonomic levels. For benthic invertebrates, data analyses are standardized according to an operative list of taxa (SEPA 2007). There are a number of benefits in having the data host in the same organization as the data producer. For example, methodological changes are well documented and artifacts related to for example instrument drift are well understood. Perhaps the best example of this is the instrument drift that occurred with low-level phosphorus measurements in the early 1990s. As the data are well scrutinized, it was possible to identify and document this problem (Sonesten and Engblom 2001).

The data collected in the Swedish surface water monitoring program play a key role in past and present national and international environmental commitments including Swedish environmental objectives, critical load assessments, and many aspects of EU legislation, such as the Water Framework Directive, Habitats Directive, and Nitrate Vulnerable Zone Directive. There are good reasons to believe that the long-term Swedish surface water monitoring program will contribute to future commitments also.

## The future

While long-term monitoring is vital for generating new scientific knowledge and crucial for environmental management and sustainability, its value has been questioned for a number of reasons. Long-term monitoring has been criticized as being unscientific, expensive, and wasteful (summarized in Lovett et al. (2007)). However, long-term monitoring is often the only means of establishing the efficacy of costly pollution control measures or for predicting regime shifts and tipping points. Furthermore, long-term monitoring data are critical for the empirical testing of resilience theory (Angeler et al. 2014).

A large number of models have been developed lately that can make predictions about unsampled locations in space and time, but the credibility of these predictions is ultimately dependent on the amount of monitoring data used for model calibration (Larssen et al. 2007). Modeling should not be seen as a substitute for a well-designed and well-supported monitoring program, but rather as a way to make better use of monitoring data.

There are repeated suggestions that monitoring programs be “rationalized” or reduced in scope. If this is to be done, it must be performed in a statistically well-informed manner (Levine et al. 2014), so as to ensure that the questions for which the monitoring was designed to address can still be answered.

New data sources such as advanced remote sensing, genetic barcoding, and high frequency measurements all show great promise for increasing our understanding of fresh waters. These new methods must be seen as a complement to, not a replacement for, long-term monitoring. Long-term monitoring data are one of the best resources for assessing climate change impacts and they also provide an invaluable resource for “ground-truthing” novel data collection methods, including crowd sourcing and citizen science.

There are opportunities to improve the Swedish national monitoring program. The representativeness of the Trend Lake program could be further improved by on-going replacement of acid sensitive lakes with better buffered lakes, since the focus has partly been moved from acidification to effects of climate change (Fölster et al. 2014). The lack of pre-impact data is one general problem with long-term monitoring. For example, Holmgren (2014) has illustrated some of the difficulties of assessing recovery when baseline data are absent. These difficulties can be addressed, in part with paleoecological techniques based on lake sediments. Sediment cores are a valuable source of long-term information about lake water quality which could be better incorporated into the national monitoring program, both for the identification of reference conditions (Norberg et al. 2008) and for improved scientific understanding (Hu and Huser 2014). Better connections between the national monitoring program database and other Swedish model and observational data sources would facilitate more analyses such as the one performed by Valinia et al. (2014), which linked long-term water chemistry observations to fish presence/absence data and modeled acidification trends.

Curtis et al. (2014) have noted that the two key challenges for scientists, policy makers, and other stakeholders are to identify the trajectory of change in human-impacted surface waters and to define ecologically meaningful endpoints. The latter is of particular importance for setting targets for phosphorus concentrations when historical loads have increased phosphorus levels in soils and lake sediments that will take many decades for a full recovery to good ecological status (Jarvie et al. 2013). Likewise, full recovery from surface water acidification is not likely to occur in the near future due to acidification of the soils in the catchments (Futter et al. 2014). High quality, long-term monitoring data can both identify historical trajectories, and if used appropriately suggest ecologically meaningful endpoints.

The interplay between the scientific community and the evolving needs of society has in the last five decades given rise to today’s Swedish national monitoring program, i.e., comprising some 50 years of time series of water chemistry and biological measurements. These well-used time series are invaluable for setting reference conditions and tracking the recovery of lake and streams ecosystems from regional impacts such as acidification (Futter et al. 2014), diffuse nutrient leaching (Ulen and Fölster 2007), and the on-going browning of surface waters (Temnerud et al. 2014). Furthermore, as mandated by the European WFD, these sites constitute a spatial reference network “For spatially based type-specific biological reference conditions, Member States shall develop a reference network for each surface water body type.“(EC 2000). Despite its long tradition of monitoring surface water quality, Sweden was recently criticized by the European Commission (EC) for classifying the ecological status of many water bodies solely using expert opinion, i.e., without biological or physicochemical measures. Classifying all water bodies (ca. 100 000 lakes and 500 000 km streams) based exclusively on measurements is an enormous challenge. One possible solution is to place more focus on probability sampling of water chemistry in combination with known relationships between water chemistry and biota. For example, the lake survey program can be used for classifying large populations of lakes deemed to be minimally impacted by anthropogenic activities, while water bodies judged to be at risk of failing to achieve good ecological status and lakes known to have less than good status can be the main focus of biological monitoring efforts.

Another issue raised by the EC was the large amount of regional monitoring done within recipient control programs. The Commission pointed out that it was often not possible to classify sites according to WFD criteria due to the quality and frequency of sampling. With strong inputs from the scientific community, Sweden is once again revising national and regional monitoring programs so as to meet changing societal demands. For example, it is anticipated that more stringent controls, such as WFD compliant sampling methodologies and certified laboratories, will be required when implementing the polluter pays principle.

## Concluding remarks

The gradual development of monitoring in cooperation between scientists and authorities is an example of 50 years of adaptive monitoring (Lindenmayer and Likens 2009, 2010). To date, the program has been able to secure adequate long-term funding. The program was originally based on well-formulated, tractable questions related to the effects of phosphorus from wastewater treatment plants on lake trophic status. As this question was addressed, new questions related to acidification (Fölster and Wilander 2002), mercury contamination (Eklöf et al. 2012), and agricultural runoff (Ulen and Fölster 2007) resulted in the refinement of the monitoring programs. Sampling is based on sound statistical principles, and there are well-formulated protocols for data collection and sample handling following existing international standards. Data are collected and analyzed in collaboration with other agencies, scientists, and stakeholders. The web-based information system ensures that data are readily available and widely used by researchers in Sweden and elsewhere. The revisions of the program over the years were driven by societal need for new knowledge and based on a scientific understanding of ecosystem function and statistical evaluation. It is hoped that the continued relevance of the Swedish national surface water monitoring program for societally relevant questions in Sweden, Europe, and elsewhere will help to ensure its growth and survival in the future.
